# Akkermansia Muciniphila Attenuates TNF-α-Induced Injury in Human Primary Colonic Epithelial Cells with Ferroptosis-Associated Redox Remodeling

**DOI:** 10.3390/microorganisms14102393

**Published:** 2026-10-19

**Authors:** Kaiyan Wei, Shuhua Zhuo, Yanjun Lin, Lin Zhang, Yijuan Liu

**Affiliations:** 1 Department of Gastroenterology, The First Affiliated Hospital, Fujian Medical University, Fuzhou 350005, China; 2 Department of Gastroenterology, National Regional Medical Center, Binhai Campus of The First Affiliated Hospital, Fujian Medical University, Fuzhou 350212, China; 3 Clinical Research Center for Liver and Intestinal Diseases of Fujian Province, The First Affiliated Hospital, Fujian Medical University, Fuzhou 350005, China; 4 The First Clinical Medical College, Fujian Medical University, Fuzhou 350005, China; 5 Department of Gastrointestinal Surgery, The First Affiliated Hospital, Fujian Medical University, Fuzhou 350005, China

**Keywords:** Akkermansia muciniphila, ulcerative colitis, epithelial injury, human primary colonic epithelial cells, TNF-α, ferroptosis-associated redox remodeling, erastin, GPX4, SLC7A11, HES/HOX

## Abstract

Akkermansia muciniphila (AKK) is linked to intestinal barrier homeostasis, but whether live AKK can directly protect human colonic epithelium during a defined cytokine insult remains less clear. We tested this question in human primary colonic epithelial cells (HCoEpiC). Cells were pretreated with live AKK at different multiplicities of infection (MOIs) before tumor necrosis factor-α (TNF-α) exposure; erastin (1.76 µM) was used to increase ferroptosis pressure. Cell viability, apoptosis, interleukin-6 (IL-6)/interleukin-1β (IL-1β) concentrations, and selected ferroptosis-associated redox and epithelial-state proteins were measured by the Cell Counting Kit-8 (CCK-8) assay, flow cytometry, enzyme-linked immunosorbent assay (ELISA), and Western blotting. TNF-α reduced viability, increased apoptosis, and raised IL-6 and IL-1β concentrations. AKK pretreatment mitigated these changes, with the clearest effect at MOI 100. AKK also increased glutathione peroxidase 4 (GPX4) and solute carrier family 7 member 11 (SLC7A11), reduced acyl-CoA synthetase long-chain family member 4 (ACSL4) and NAD(P)H quinone dehydrogenase 1 (NQO1), and increased hes family bHLH transcription factor 1 (HES1), homeobox A13 (HOXA13), and homeobox B13 (HOXB13). Erastin partly weakened the AKK-associated improvements in viability, apoptosis, cytokine levels, and protein expression. These findings show that live AKK attenuates TNF-α-induced injury in primary human colonic epithelial cells. The protective pattern is accompanied by ferroptosis-associated redox remodeling and recovery of HES/HOX-associated epithelial-state markers, supporting further testing in ulcerative colitis (UC)-relevant epithelial injury models.

## 1. Introduction

Ulcerative colitis (UC) reflects a recurring injury-repair problem at the mucosal surface. Current guidelines place mucosal healing and durable disease control at the center of treatment, but primary non-response, loss of response, and residual mucosal injury still occur during biologic therapy, including anti-TNF-α treatment [1,2,3,4]. These clinical limits support epithelial resilience as an adjunctive target, particularly for approaches intended to reinforce epithelial recovery alongside cytokine blockade.

The microbiota can influence that epithelial compartment. Akkermansia muciniphila (AKK) is a mucin-associated anaerobe positioned close to the epithelial surface, and it has been linked to barrier function, immune tone, and metabolic homeostasis [5,6]. Experimental studies show that AKK-derived signals can regulate barrier programs and wound repair [7,8]. These effects depend on biological context, including strain background, viability, processing, dose, and host setting. Evidence from animals, mixed microbial communities, or systemic immune readouts therefore does not fully answer whether live AKK can act directly on primary human colonic epithelial cells during TNF-α stress.

Ferroptosis offers a focused way to examine that response. This iron-dependent regulated cell-death program is driven by lipid peroxidation and restrained by the system Xc−/glutathione/GPX4 axis [9]. Under inflammatory stress, a shift in GPX4, SLC7A11, and ACSL4 can indicate whether epithelial cells are moving toward or away from a lipid-peroxidation-prone state. We included NQO1 as a companion readout of oxidative-stress tone, so that the protein panel could capture both ferroptosis-associated defense and the surrounding redox response.

Epithelial injury also changes the state of surviving cells. HES1, a Notch-responsive transcriptional effector, is involved in intestinal epithelial fate allocation and renewal [10]. We measured HES1 with HOXA13 and HOXB13 because HOX programs help define regional identity along the gut, and HOXA13/HOXB13 have been used as posterior hindgut or colonic epithelial identity markers in human colonic organoid systems [10,11,12]. Here, these proteins were treated as epithelial-state markers rather than as proven drivers of the AKK response.

Using commercially available human primary colonic epithelial cells (HCoEpiC), we asked whether live AKK could reduce TNF-α-induced epithelial injury in a defined human-cell system. Cells were exposed to AKK at two multiplicities of infection before TNF-α injury induction. Erastin was used as a ferroptosis-pressure challenge to test whether the AKK-associated phenotype was sensitive to increased pressure on the system Xc−/GPX4 redox axis.

## 2. Materials and Methods

### 2.1. Bacterial Strain, Cells, and Major Reagents

Human primary colonic epithelial cells (HCoEpiC) and the matched serum-free complete culture medium were purchased from iCell Bioscience Inc. (Shanghai, China). The cells were supplied as commercially available primary cells, and donor-level clinical information was not available to the authors. Akkermansia muciniphila (AKK; BNCC341917) was obtained from BeNa Culture Collection (Beijing, China). AKK was cultured at 37 °C under anaerobic conditions in brain heart infusion (BHI) broth (HB8927; Hopebio, Qingdao, China) supplemented with mucin. To prepare the medium, 19.25 g BHI powder and 1 g mucin were dissolved in 500 mL distilled water, sterilized at 115 °C for 15 min, cooled, and stored at 4 °C before use. AKK was subcultured at a 5% inoculation ratio; bacterial cultures from passages 3–4 were used for epithelial-cell co-culture after Gram staining and optical density at 600 nm (OD600) measurement. Gram staining under light microscopy showed uniformly stained Gram-negative oval bacteria, consistent with the expected morphology of AKK. Recombinant human TNF-α used for injury induction was purchased from Wuhan Elabscience Biotechnology Co., Ltd. (Wuhan, China). The ferroptosis inducer erastin was purchased from MedMol, a brand of Shanghai Yuanye Bio-Technology Co., Ltd. (Shanghai, China; lot No. S22IS226768).

Immediately before each co-culture experiment, AKK inocula were quantified from the cultured bacterial suspension. OD600 was measured at 600 nm, and one OD600 unit was taken to correspond to 3.88 × 10^7^ colony-forming units (CFU)/mL. Bacteria were collected by centrifugation at 8000 rpm for 5 min using a 5424R refrigerated centrifuge (Eppendorf, Hamburg, Germany) and diluted to the required working concentration. For each preparation, the volume of the original bacterial suspension (Vx) needed for a final working volume of 0.6 mL was calculated as OD600 × (3.88 × 10^7^ CFU/mL) × Vx = C × V, where C is the target bacterial concentration and V is the target working volume. The calculation was repeated with the measured OD600 value for each newly prepared AKK suspension.

**Figure 1:**
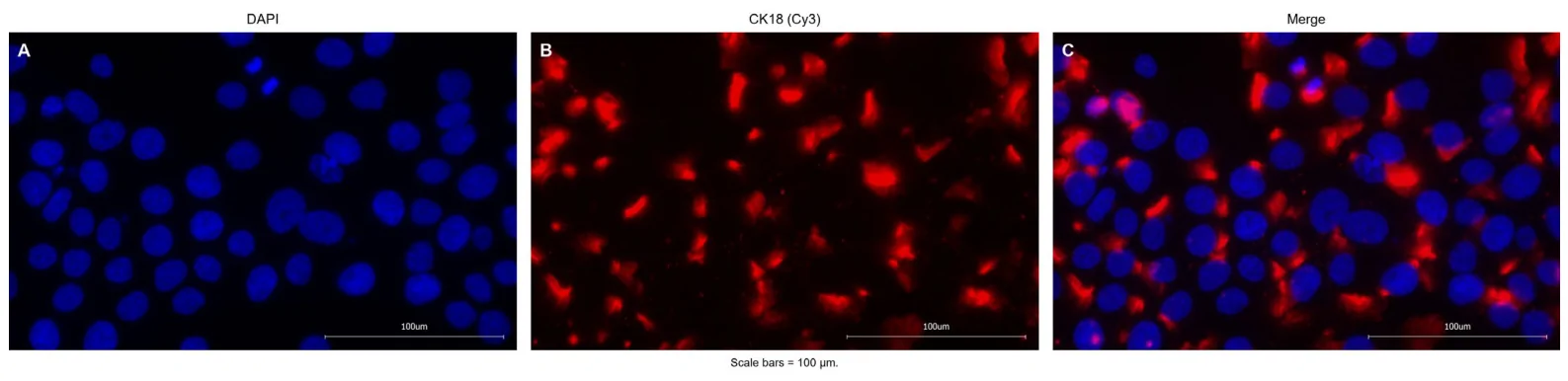
Immunofluorescence confirmation of the epithelial phenotype of HCoEpiC. (**A**) DAPI nuclear staining (blue); (**B**) CK18 immunofluorescence (Cy3, red); (**C**) merged image. Scale bars = 100 µm.

To provide visual confirmation of the epithelial phenotype, HCoEpiC were assessed by cytokeratin 18 (CK18) immunofluorescence. Cells grown on coverslips were fixed and incubated with a rabbit anti-human CK18 primary antibody (1:200), followed by a Cy3-labeled goat anti-rabbit IgG secondary antibody (1:200); nuclei were counterstained with 4′,6-diamidino-2-phenylindole (DAPI). Representative images are shown in Figure 1.

Cell-function assay reagents included a Cell Counting Kit-8 (CCK-8) cell viability assay kit, an Annexin V-fluorescein isothiocyanate (FITC)/propidium iodide (PI) apoptosis detection kit, and human cytokine enzyme-linked immunosorbent assay (ELISA) kits. The ELISA kits for IL-6 (E-EL-H6156), IL-1β (E-EL-H0149c), and TNF-α (E-EL-H0109c) were supplied by Elabscience Biotechnology Co., Ltd. (Wuhan, China).

For Western blotting, the primary antibody panel included mouse anti-GPX4 (Proteintech Group, Wuhan, China; 67763-1-Ig; 1:2000), rabbit anti-SLC7A11 (OmnimAbs, Alhambra, CA, USA; OM111565; 1:1000), rabbit anti-ACSL4 (Affinity Biosciences, Cincinnati, OH, USA; DF12141; 1:2000), rabbit anti-HES1 (Affinity Biosciences, Cincinnati, OH, USA; DF7569; 1:2000), rabbit anti-HOXA13 (Bioss, Beijing, China; BS-12244R; 1:1000), rabbit anti-HOXB13 (ABclonal, Wuhan, China; A15779; 1:1000), mouse anti-NQO1 (Proteintech Group, Wuhan, China; 67240-1-Ig; 1:20,000), and mouse anti-β-actin (TransGen Biotech, Beijing, China; HC201; 1:2000). Horseradish peroxidase (HRP)-conjugated goat anti-mouse IgG (Servicebio, Wuhan, China; GB23301; 1:2000) and HRP-conjugated goat anti-rabbit IgG (Servicebio, Wuhan, China; GB23303; 1:2000) were used as secondary antibodies. This antibody panel covered ferroptosis-associated redox proteins, epithelial-state markers, and the β-actin loading control. Radioimmunoprecipitation assay (RIPA) lysis buffer was used for protein extraction, the bicinchoninic acid (BCA) assay for protein quantification, polyvinylidene difluoride (PVDF) membranes for transfer, and enhanced chemiluminescence (ECL) substrate for signal detection. Primary antibodies were incubated overnight at 4 °C, HRP-conjugated secondary antibodies were incubated for 2 h at room temperature, and band intensity was normalized to β-actin.

Major equipment included a humidified 5% CO_2_ cell culture incubator (Shanghai Yiheng Scientific Instrument Co., Ltd., Shanghai, China), a NovoCyte flow cytometer (Agilent Technologies, Santa Clara, CA, USA), a SuPerMax 3100 multifunctional microplate reader (Shanghai Flash Spectrum Biological Technology Co., Ltd., Shanghai, China), a Tanon-5200 chemiluminescence imaging system (Tanon Science & Technology Co., Ltd., Shanghai, China), a 5424R refrigerated centrifuge (Eppendorf, Hamburg, Germany), and an anaerobic workstation.

### 2.2. Cell Culture and Experimental Grouping

After recovery, HCoEpiC were cultured at 37 °C in a humidified incubator (Shanghai Yiheng Scientific Instrument Co., Ltd., Shanghai, China) containing 5% CO_2_. Cells were passaged when confluence reached 80–90%, and cells from passages 3–6 with stable growth and no evidence of senescence or contamination were used for all in vitro functional and molecular experiments. Two experimental systems were run in parallel: a graded AKK-dose system and an erastin-intervention system. MOI was defined as the estimated number of viable AKK bacterial cells added per epithelial cell at the start of AKK exposure. Before each co-culture experiment, epithelial cells were counted, the AKK suspension was quantified using the OD600-to-CFU conversion described above, and the bacterial suspension was diluted to generate MOI 50 or MOI 100. Thus, each AKK dose was determined from both the measured bacterial density and the epithelial-cell number at treatment.

System 1: AKK dose-gradient intervention groups

Control group: untreated normal culture control;

TNF-α model group: 30 ng/mL TNF-α alone;

TNF-α + AKK-Low group: TNF-α + AKK at MOI = 50;

TNF-α + AKK-High group: TNF-α + AKK at MOI = 100.

System 2: erastin-intervention groups

TNF-α model group;

TNF-α + erastin group: TNF-α + 1.76 µM erastin (equivalent to 960 ng/mL, calculated using a molecular weight of 547.04 g/mol) pretreatment;

TNF-α + AKK-High group: TNF-α + AKK at MOI = 100;

TNF-α + AKK + erastin combined-treatment group.

The standardized treatment sequence was as follows. AKK cultures were recovered, passaged to passages 3–4, checked by Gram staining, quantified by OD600, and diluted to the required MOI before addition to HCoEpiC cultures. In all AKK-containing groups, the prepared live-AKK suspension was co-incubated with HCoEpiC for 24 h. For TNF-α-induced injury, 30 ng/mL recombinant TNF-α was then added for 24 h. In the AKK + erastin combined-treatment group, cells were exposed to AKK for 24 h, treated with 1.76 µM erastin (equivalent to 960 ng/mL, calculated using a molecular weight of 547.04 g/mol) for 1 h, and then treated with 30 ng/mL TNF-α for 24 h. The erastin concentration was selected from the preliminary dose screen. Among the tested range (80–1280 ng/mL), 960 ng/mL produced a clear but not maximal reduction in viability, leaving a dynamic range to evaluate whether AKK-associated protection was weakened by increased ferroptosis pressure. At the endpoint, culture supernatants and cell pellets were collected for cytokine, apoptosis, and protein-expression assays.

### 2.3. CCK-8 Cell Viability Assay

HCoEpiC were seeded into 96-well plates at a density of 1 × 10^3^ cells per well. After complete attachment, cells were treated according to the experimental grouping described above. CCK-8 working solution (10 µL) was added to each well, followed by incubation at 37 °C for 2 h protected from light. Absorbance at 450 nm (OD450) was measured using a SuPerMax3100 multifunctional microplate reader (Shanghai Flash Spectrum Biological Technology Co., Ltd., Shanghai, China). Five technical wells were set for each group in each experiment, and the full experiment was independently repeated three times. Cell viability was normalized to the control group, which was defined as 100%. Because live bacterial preparations can influence colorimetric viability assays, CCK-8 findings were interpreted together with apoptosis, cytokine, and Western blot endpoints rather than as a stand-alone measure.

### 2.4. Flow-Cytometric Quantification of Apoptosis

At least 1 × 10^6^ cells were collected from each group and washed twice with precooled sterile phosphate-buffered saline (PBS). The cell pellet was resuspended in 1× binding buffer and stained with Annexin V-FITC and PI for 10 min at room temperature in the dark. Fluorescence signals were acquired using a NovoCyte flow cytometer (Agilent Technologies, Santa Clara, CA, USA), and total apoptotic cells, including early and late apoptotic cells, were calculated as a percentage of total cells. Three independent biological replicates were included for each group.

### 2.5. ELISA Measurement of Pro-Inflammatory Cytokines in Culture Supernatants

Culture supernatants from each group were collected at the endpoint and centrifuged under cold conditions to remove cell debris. Standard gradients, wash buffer, and chromogenic substrate were prepared according to the ELISA kit instructions, and absolute cytokine concentrations were interpolated from standard curves. IL-6, IL-1β, and TNF-α concentrations were measured using Elabscience kits E-EL-H6156, E-EL-H0149c, and E-EL-H0109c, respectively, and absorbance values were acquired using a SuPerMax 3100 multifunctional microplate reader (Shanghai Flash Spectrum Biological Technology Co., Ltd., Shanghai, China). Three biological replicates were analyzed for each group, and all measurements were performed according to the manufacturer’s protocols. For TNF-α, the assay measured extracellular TNF-α concentration in supernatants from a system receiving recombinant TNF-α; therefore, this endpoint was interpreted as extracellular TNF-α dynamics under the experimental conditions rather than as direct evidence of de novo epithelial TNF-α production.

### 2.6. Western Blot Analysis of Target Protein Expression

Total cellular protein was extracted using RIPA lysis buffer on ice, and protein concentrations were determined using the BCA method. Equal amounts of protein were denatured in boiling water for 5 min, separated by sodium dodecyl sulfate–polyacrylamide gel electrophoresis (SDS–PAGE), and wet-transferred onto PVDF membranes. After blocking with 5% nonfat milk for 1 h at room temperature, membranes were incubated with the primary antibodies listed above overnight at 4 °C with gentle shaking. Membranes were washed three times with Tris-buffered saline containing Tween-20 (TBST) for 10 min each and then incubated with the corresponding HRP-conjugated secondary antibody for 2 h at room temperature. After additional washing, ECL chemiluminescent substrate was applied, and protein bands were captured using a Tanon-5200 chemiluminescence imaging system (Tanon Science & Technology Co., Ltd., Shanghai, China). Band intensities were quantified using ImageJ software (version 1.52a, U.S. National Institutes of Health, Bethesda, MD, USA), normalized to β-actin, and expressed as relative protein levels. Western blot experiments were independently repeated three times.

### 2.7. Statistical Analysis

All quantitative data are presented as mean ± standard deviation (mean ± SD). A biological replicate was defined as an independently prepared cell culture subjected to the full treatment and endpoint-collection procedure, whereas a technical replicate referred to repeated wells or repeated measurements within the same experimental run. The replicate structure for each endpoint is stated in the corresponding method section, figure legend, and Supplementary Table S2. Statistical analyses and graphing were performed using GraphPad Prism version 9.5.1 (GraphPad Software, Boston, MA, USA). Data distribution was assessed using the Shapiro–Wilk test, and homogeneity of variance was assessed using Bartlett’s test where applicable. Independent-sample *t* tests were used for comparisons between two groups. Overall differences among multiple groups were analyzed by one-way analysis of variance (ANOVA), followed by Tukey’s post hoc test for pairwise comparisons. All datasets used for the reported between-group comparisons were analyzed under this parametric framework after normality and variance screening; no non-parametric alternative was applied. *p* < 0.05 was considered statistically significant.

## 3. Results

Before the treatment experiments, CK18 immunofluorescence was performed to confirm the epithelial phenotype of HCoEpiC. CK18 staining showed a cytoplasmic epithelial signal, and DAPI labeled the nuclei, supporting the epithelial identity of the cell model used in subsequent TNF-α-induced injury assays (Figure 1). AKK preparations used for intervention showed uniformly stained Gram-negative oval morphology after Gram staining, and each bacterial inoculum was quantified by OD600-based CFU estimation before co-culture.

### 3.1. AKK Dose-Dependently Alleviates TNF-α-Induced Injury in Human Primary Colonic Epithelial Cells

**Figure 2:**
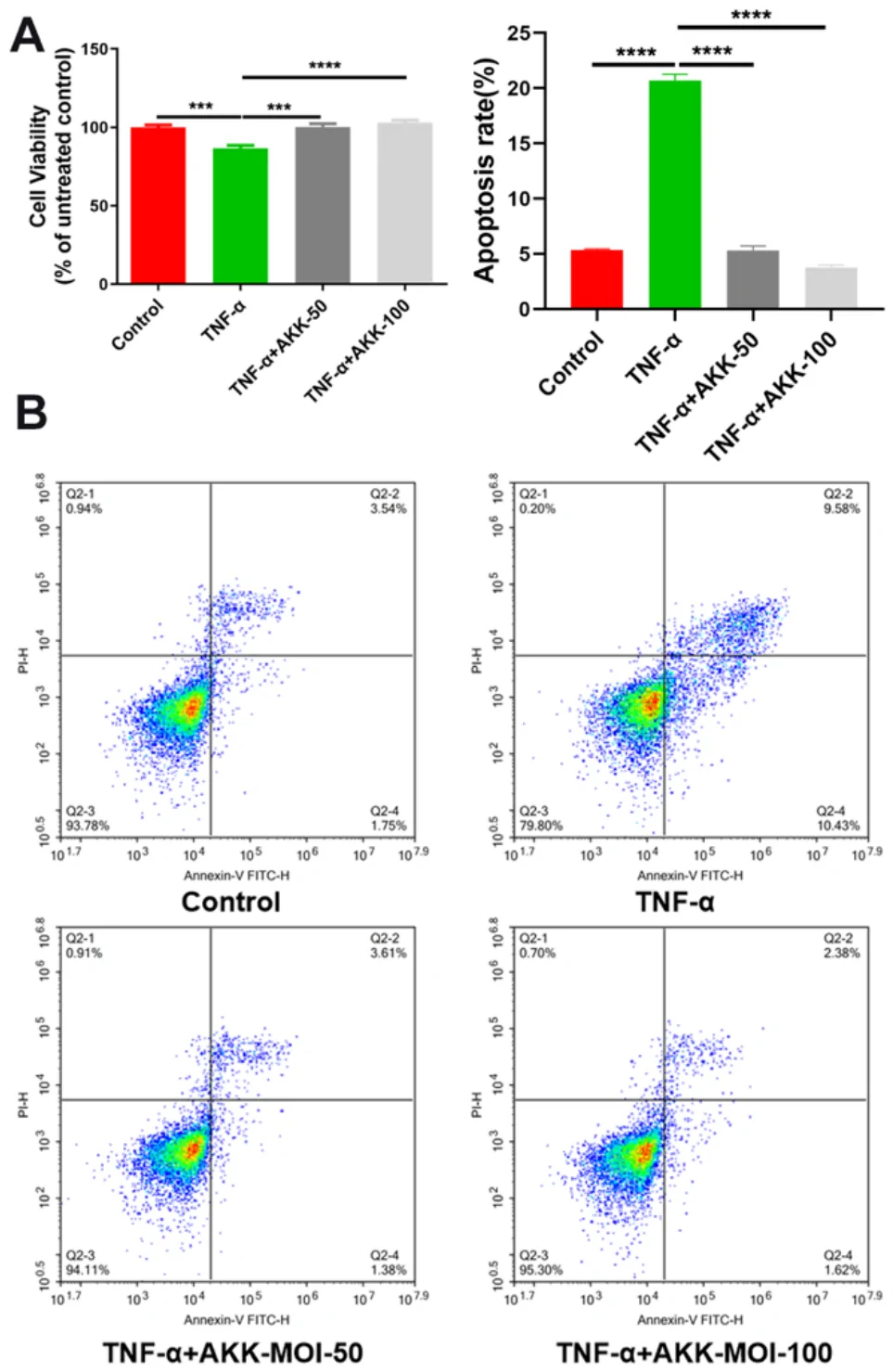
Effects of Akkermansia muciniphila (AKK) on cell viability and apoptosis in human primary colonic epithelial cells. (**A**) Cell viability was measured by the CCK-8 assay (five technical wells per experiment; three independent experiments). (**B**) Apoptosis was detected by flow cytometry (three independent experiments). Data were analyzed after normality and variance screening; no non-parametric alternative was applied. *** *p* < 0.001; **** *p* < 0.0001.

TNF-α reduced HCoEpiC viability and increased total apoptosis compared with the control group (*p* < 0.05). AKK improved both endpoints, with a clearer effect at MOI 100 than at MOI 50 (*p* < 0.05). Because MOI values were calculated from the measured OD600-derived bacterial density and the epithelial-cell count, these results compare two predefined AKK exposure levels rather than an unspecified bacterial inoculum. Thus, in a primary human epithelial monoculture without immune cells or additional commensal organisms, live AKK attenuated TNF-α-mediated viability loss and apoptotic activation (Figure 2).

### 3.2. AKK Lowers TNF-α-Induced IL-6 and IL-1β Release by Colonic Epithelial Cells

**Figure 3:**
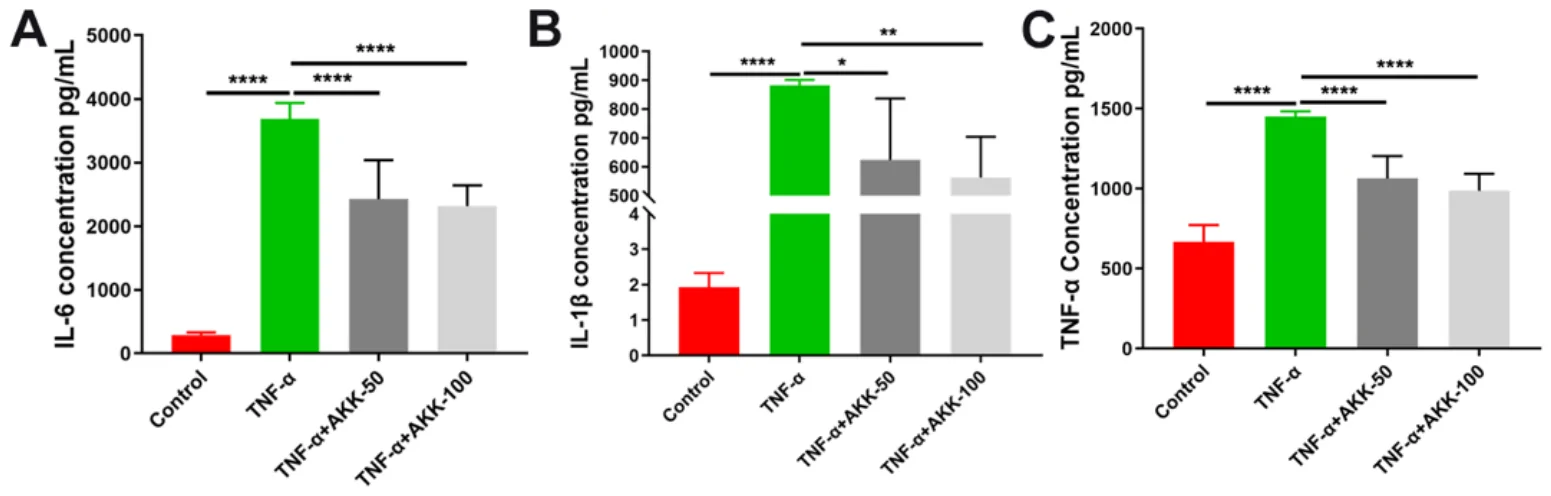
Effects of Akkermansia muciniphila (AKK) on IL-6, IL-1β, and extracellular TNF-α concentrations in human primary colonic epithelial cell supernatants. (**A**) IL-6 concentration measured using ELISA kit E-EL-H6156 (three biological replicates). (**B**) IL-1β concentration measured using ELISA kit E-EL-H0149c (three biological replicates). (**C**) TNF-α concentration measured using ELISA kit E-EL-H0109c in the recombinant TNF-α-treated culture system (three biological replicates). TNF-α values were interpreted as extracellular TNF-α dynamics under the experimental conditions. Data were analyzed after normality and variance screening; no non-parametric alternative was applied. * *p* < 0.05; ** *p* < 0.01; **** *p* < 0.0001.

TNF-α exposure increased IL-6 and IL-1β concentrations in the culture supernatant compared with the control group (*p* < 0.05). ELISA measurements were performed with target-specific Elabscience kits and standard curves using three biological replicates per group. Graded AKK co-culture lowered both cytokines, with the most consistent reduction at MOI 100. Supernatant TNF-α also varied across groups. Because recombinant TNF-α was the injury stimulus, this measurement is best interpreted as extracellular TNF-α dynamics under the culture conditions, not as a stand-alone measure of newly secreted epithelial TNF-α. IL-6 and IL-1β therefore provide the clearest endogenous cytokine evidence that AKK reduced epithelial inflammatory output under TNF-α-induced stress (Figure 3).

### 3.3. AKK Modulates Ferroptosis-Associated Redox Proteins and HES/HOX-Related Epithelial-State Markers

**Figure 4:**
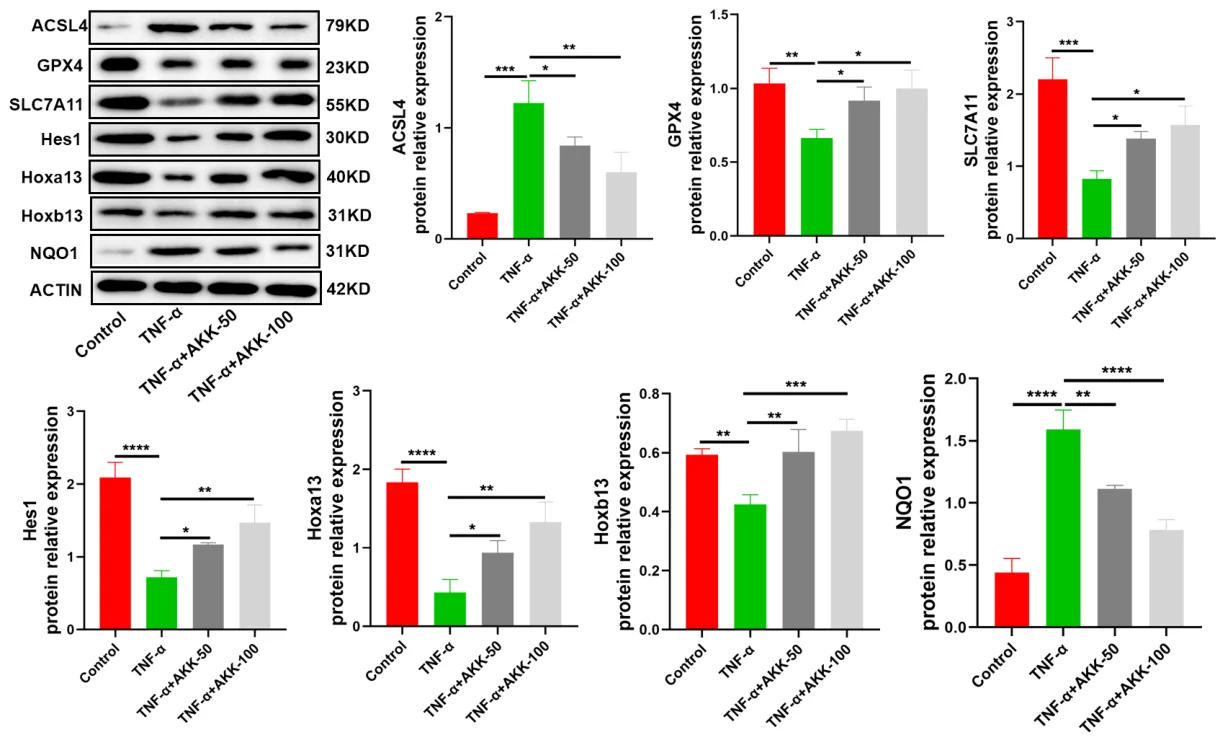
Effects of Akkermansia muciniphila (AKK) on ferroptosis-associated redox proteins and HES/HOX-associated epithelial-state markers in human primary colonic epithelial cells. Protein expression levels of ACSL4, GPX4, SLC7A11, HES1, HOXA13, HOXB13, and NQO1 were assessed by Western blotting (three independent experiments). Data were analyzed after normality and variance screening; no non-parametric alternative was applied. * *p* < 0.05; ** *p* < 0.01; *** *p* < 0.001; **** *p* < 0.0001.

Western blotting showed that TNF-α lowered GPX4 and SLC7A11 and increased ACSL4 and NQO1. HES1, HOXA13, and HOXB13 were also reduced (*p* < 0.05). AKK moved these protein changes back toward the control pattern, most clearly at MOI 100. The matched recovery of redox proteins and epithelial-state markers suggests that AKK treatment was associated with a less ferroptosis-prone redox profile and a more recovery-compatible epithelial state (Figure 4).

### 3.4. Erastin Weakens the AKK-Associated Protective Phenotype

**Figure 5:**
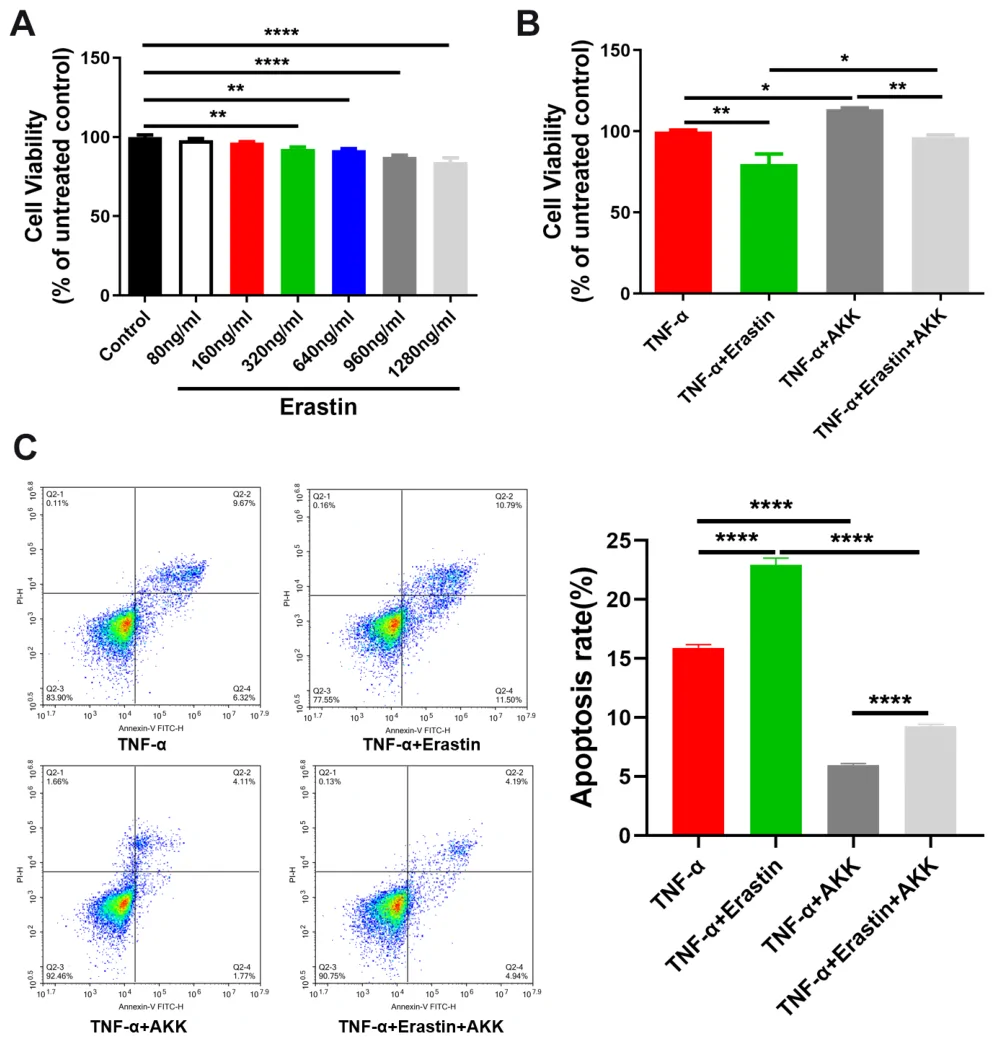
The system Xc− inhibitor erastin weakens the AKK-associated protective phenotype in human primary colonic epithelial cells. (**A**) Optimization of erastin concentration by the CCK-8 assay (five technical wells per experiment; three independent experiments). (**B**) Cell viability in each treatment group measured by the CCK-8 assay (five technical wells per experiment; three independent experiments). (**C**) Apoptosis in each treatment group detected by flow cytometry (three independent experiments). Data were analyzed after normality and variance screening; no non-parametric alternative was applied. * *p* < 0.05; ** *p* < 0.01; **** *p* < 0.0001.

Erastin pretreatment alone deepened the loss of viability and increase in apoptosis caused by TNF-α. When erastin was combined with high-dose AKK, the AKK-related viability benefit was reduced. Erastin also blunted the AKK-associated decrease in IL-6 and IL-1β and shifted the protein profile back toward lower GPX4/SLC7A11/HES1/HOXA13/HOXB13 and higher ACSL4/NQO1. These findings show that the AKK-associated epithelial phenotype is sensitive to increased ferroptosis pressure. They do not, by themselves, prove ferroptosis as the sole mediator; direct lipid-peroxidation assays and inhibitor-rescue experiments would be needed for that conclusion (Figure 5).

## 4. Discussion

This study shows that live AKK protected primary human colonic epithelial cells from TNF-α-induced injury in a defined epithelial system. TNF-α lowered viability, increased apoptosis, and raised the IL-6/IL-1β concentrations; AKK moved each endpoint in the protective direction, with the clearest response at MOI 100. This matters for UC research because mucosal healing depends on epithelial recovery, yet incomplete response and residual injury still occur during anti-TNF-based treatment [2,3,4]. By isolating epithelial cells from immune and microbiota-wide inputs, the present study adds a human epithelial layer to AKK research rather than another whole-animal or association-based observation.

These findings fit the current AKK literature but narrow the question to epithelial autonomy. AKK-derived signals can activate barrier programs and promote epithelial wound repair [7,8]. Other work shows that an AKK phospholipid can shape immune homeostasis, and that pasteurization, autoclaving, and viability can change host responses [13,14,15]. IBD-focused studies also connect AKK with barrier protection, microbial remodeling, and oxidative-stress control [16,17,18]. Our data extend this work by showing that live AKK can reduce a TNF-α injury phenotype even when immune cells and a complex microbial community are absent.

The redox-protein data point to ferroptosis-associated regulation as one contributor to this protection. TNF-α lowered GPX4/SLC7A11 and increased ACSL4, consistent with weaker lipid-peroxide detoxification and higher ferroptosis vulnerability [9,19]. AKK largely reversed this pattern, and erastin partly removed the gains in viability, apoptosis control, cytokine reduction, and protein expression. This pharmacological result links the ferroptosis-associated signal to the functional response of the cells. The interpretation is also consistent with UC and colitis studies that connect epithelial ferroptosis with barrier injury and therapeutic response, including NEDD4L- and HIF-1α/GPX4-related findings [20,21,22] and intervention studies in which ferroptosis control accompanies improved epithelial integrity [23,24,25]. NQO1 is best interpreted here as an oxidative-stress response enzyme; its decline after AKK treatment tracks with lower redox pressure rather than serving as a standalone ferroptosis marker [26]. Overall, the coordinated recovery of GPX4/SLC7A11, reduction in ACSL4, and lower NQO1 expression indicate that AKK shifted injured epithelial cells toward a less ferroptosis-prone redox state.

The HES/HOX data add a repair-state dimension. Epithelial recovery requires the survival of cells and re-establishment of a stable epithelial program. HES1 belongs to Notch-responsive intestinal programs involved in epithelial cell-fate allocation and renewal [10]. HOX proteins help define regional identity along the gut, and HOXA13/HOXB13 have been used as posterior hindgut or colonic identity markers in human colonic differentiation systems [11,12]. In this study, HES1, HOXA13, and HOXB13 rose together with higher viability, lower apoptosis, lower IL-6/IL-1β concentrations, and a less ferroptosis-prone protein pattern. This paired movement supports a recovery-associated epithelial state after AKK treatment, without requiring a claim that HES/HOX lies upstream of ferroptosis.

The cytokine data fit the same pattern. AKK lowered IL-6 and IL-1β after TNF-α exposure, indicating that live AKK reduced epithelial inflammatory output as well as cell-death susceptibility. The TNF-α ELISA result needs a narrower reading because recombinant TNF-α was added to the medium. It reflects extracellular TNF-α dynamics in this culture system, not direct proof of new epithelial TNF-α synthesis. For that reason, IL-6 and IL-1β are the stronger endogenous cytokine endpoints, and they align with the viability, apoptosis, and protein data.

Several recent studies make this epithelial-redox interpretation plausible. AKK-derived propionate protects the intestine from irradiation-associated injury [27]. AKK-enriched or AKK-containing interventions improve colitis-related outcomes, including barrier and innate immune features, in vivo [28,29]. More recent work links AKK to ferroptosis signaling in colitis and to FXR/XBP1 regulation [30,31]. In addition, UC-oriented studies link postbiotic signaling, lactate transport, selenium, and butyrate to ferroptosis control and GPX4-centered epithelial defense [32,33,34,35]. Together, these studies frame the present work. In one primary human epithelial setting, live AKK reduced a defined TNF-α injury response, erastin weakened that protection, and the same samples showed a coordinated GPX4/SLC7A11-ACSL4/NQO1 shift with the recovery of HES1, HOXA13, and HOXB13. This gives future AKK-preparation work a focused human-cell assay before moving to organoids, epithelial-immune co-cultures, or mucosal models.

This study also has clear experimental boundaries. The monoculture design isolated epithelial responses but did not include a mucus layer, immune or stromal cells, vascular signals, anaerobic-aerobic gradients, or whole-microbiota interactions. Ferroptosis involvement was supported by GPX4/SLC7A11, ACSL4, and NQO1 changes together with erastin sensitivity; direct lipid ROS, labile iron, GSH/MDA, and ferroptosis-inhibitor rescue assays would sharpen the pathway map. The original design used live AKK only and did not include AKK-only, heat-killed/pasteurized AKK, or post-co-culture bacterial-viability controls. These boundaries do not change the observed epithelial protective phenotype, but they define the next experiments needed to separate bacterial contact, membrane molecules, phospholipids, and soluble metabolites.

## 5. Conclusions

In primary human colonic epithelial cells, live AKK reduced TNF-α-induced viability loss, apoptosis, and IL-6/IL-1β increases. The response was accompanied by the recovery of GPX4/SLC7A11, lower ACSL4/NQO1, higher HES1/HOXA13/HOXB13, and partial loss of protection after erastin exposure. These findings support AKK-associated epithelial protection linked to ferroptosis-associated redox remodeling and HES/HOX-related epithelial-state recovery in a defined human-cell model. Direct lipid-peroxidation assays, ferroptosis-inhibitor rescue, AKK-only and heat-killed AKK controls, and organoid or co-culture validation are the next steps for testing this mechanism in more complete UC mucosal systems.

## Data Availability

The data supporting the findings of this study are available from the corresponding author upon reasonable request.

## Supplementary Materials

The following supporting information can be downloaded at: https://www.mdpi.com/article/10.3390/microorganisms14102393/s1, Table S1, Experimental grouping and treatment schedule; Table S2, Endpoint assays and replicate design; original Western blot images. Representative CK18 immunofluorescence images are included as Figure 1 in the main manuscript.

## Abbreviations

UC, ulcerative colitis; AKK, Akkermansia muciniphila; HCoEpiC, human primary colonic epithelial cells; MOI, multiplicity of infection; CCK-8, cell counting kit-8; ELISA, enzyme-linked immunosorbent assay; GPX4, glutathione peroxidase 4; SLC7A11, solute carrier family 7 member 11; ACSL4, acyl-CoA synthetase long-chain family member 4; NQO1, NAD(P)H quinone dehydrogenase 1; HES1, hes family bHLH transcription factor 1; HOXA13, homeobox A13; HOXB13, homeobox B13.
