# Supplementary material for: Akkermansia Muciniphila Attenuates TNF-α-Induced Injury in Human Primary Colonic Epithelial Cells with Ferroptosis-Associated Redox Remodeling
Source: Microorganisms. 2026 Oct 19;14(10):2393. doi: 10.3390/microorganisms14102393 (PMC13707850; doi:10.3390/microorganisms14102393)
Supplement: Supplementary file 1 [file microorganisms-14-02393-s001.zip › Supplementary tables.pdf]

Supplementary Materials

Akkermansia muciniphila Attenuates TNF-α-Induced Injury in Human Primary Colonic Epithelial Cells by Suppressing Ferroptosis-Associated Responses

Table S1. Experimental grouping and treatment schedule

System	Group	TNF-α	AKK	Erastin
Dose intervention	Control	No	No	No
Dose intervention	TNF-α model	30 ng/mL, 24 h	No	No
Dose intervention	TNF-α + AKK-Low	30 ng/mL, 24 h	MOI = 50; 24 h pretreatment	No
Dose intervention	TNF-α + AKK-High	30 ng/mL, 24 h	MOI = 100; 24 h pretreatment	No
Erastin intervention	TNF-α model	30 ng/mL, 24 h	No	No
Erastin intervention	TNF-α + Erastin	30 ng/mL, 24 h	No	1.76 μM (960 ng/mL); 1 h pretreatment
Erastin intervention	TNF-α + AKK-High	30 ng/mL, 24 h	MOI = 100; 24 h pretreatment	No
Erastin intervention	TNF-α + AKK + Erastin	30 ng/mL, 24 h	MOI = 100; 24 h pretreatment	1.76 μM (960 ng/mL); 1 h pretreatment

Table S2. Endpoint assays and replicate design

Endpoint	Assay	Replicate design	Related figure
Cell viability	CCK-8	Five technical replicates; experiments repeated independently three times	Figures 2A and 5A,B
Apoptosis	Annexin V-FITC/PI flow cytometry	Three independent biological replicates	Figures 2B and 5C
Pro-inflammatory cytokines	ELISA for IL-6, IL-1β, and TNF-α	Three technical replicates per group	Figure 3
Protein expression	Western blotting	Three independent repeats; β-actin normalization	Figure 4

Note: These supplementary tables summarize the treatment design and assay structure described in the manuscript. Erastin was recorded as MedMol, lot No. S22IS226768.
